# Comparative proteomic analysis of Gib2 validating its adaptor function in *Cryptococcus neoformans*

**DOI:** 10.1371/journal.pone.0180243

**Published:** 2017-07-07

**Authors:** Gillian O. Bruni, Blake Battle, Ben Kelly, Zhengguang Zhang, Ping Wang

**Affiliations:** 1 Department of Microbiology, Immunology, and Parasitology, Louisiana State University Health Sciences Center, New Orleans, LA, United States of America; 2 College of Arts and Sciences, Loyola University New Orleans, New Orleans, LA, United States of America; 3 Department of Plant Pathology, College of Plant Protection, Nanjing Agricultural University, Nanjing, China; 4 Department of Pediatrics, Louisiana State University Health Sciences Center, New Orleans, LA, United States of America; Yonsei University, REPUBLIC OF KOREA

## Abstract

*Cryptococcus neoformans* causes often-fatal fungal meningoencephalitis in immunocompromised individuals. While the exact disease mechanisms remain elusive, signal transduction pathways mediated by key elements such as G-protein α subunit Gpa1, small GTPase Ras1, and atypical Gβ-like/RACK1 protein Gib2 are known to play important roles in *C*. *neoformans* virulence. Gib2 is important for normal growth, differentiation, and pathogenicity, and it also positively regulates cAMP levels in conjunction with Gpa1. Interestingly, Gib2 displays a scaffold protein property by interacting with a wide variety of cellular proteins. To explore Gib2 global regulatory functions, we performed two-dimensional differential gel electrophoresis (DIGE) analysis and found that *GIB2* disruption results in an increased expression of 304 protein spots (43.4%) and a decreased expression of 396 protein spots (56.6%). Analysis of 96 proteins whose expression changes were deemed significant (≥ +/- 1.5- fold) revealed that 75 proteins belong to at least 12 functional protein groups. Among them, eight groups have the statistical stringency of p ≤ 0.05, and four groups, including Hsp70/71 heat shock protein homologs and ribosomal proteins, survived the Bonferroni correction. This finding is consistent with earlier established roles for the human Gβ-like/RACK1 and the budding yeast *Saccharomyces cerevisiae* Asc1. It suggests that Gib2 could also be part of the complex affecting ribosomal biogenesis and protein translation in *C*. *neoformans*. Since eukaryotic Hsp70/71 proteins are involved in the facilitation of nascent protein folding, processing, and protection of cells against stress, we also propose that Gib2-regulated stress responses are linked to fungal virulence. Collectively, our study supports a conserved role of Gβ-like/RACK/Gib2 proteins in the essential cellular process of ribosomal biogenesis and protein translation. Our study also highlights a multifaceted regulatory role of Gib2 in the growth and pathogenicity of *C*. *neoformans*.

## Introduction

*Cryptococcus neoformans* is a basidiomycetous fungal pathogen that has a predilection for the human central nervous system causing meningoencephalitis in individuals with a compromised immune status. Virulence of the fungus is multifaceted depending on many factors, including, but not limiting to, the ability to grow at the host body temperature, elaborate the melanin pigment and a polysaccharide capsule, and produce proteinases such as ureases and phospholipases (reviewed by [1]).

Guanine nucleotide binding protein (G-protein)-mediated signal transduction pathways are one of the most important mechanisms by which eukaryotic cells sense extracellular signals and integrate them via intrinsic signals or pathways, such as cAMP or the MAP kinase pathways (reviewed in [2]). In *C*. *neoformans*, three Gα protein subunits Gpa1, Gpa2, and Gpa3 function in two distinct signaling pathways [3]. Gpa1, adenylyl cyclase Cac1, protein kinase A regulatory subunit Pkr1 and catalytic subunit Pka1, and Cac1 associated protein Aca1 govern a cAMP-dependent signaling pathway to directly impact fungal pathogenicity [4,5,6]. Meanwhile, Gpa2 and Gpa3 function in a distinct pathway(s) to regulate pheromone responsive mating and differentiation that is also to certain degree linked to virulence [3,7,8].

To understand mechanisms by which Gα Gpa1 exhibits its regulatory function in *C*. *neoformans*, we identified Gib2 as an atypical Gβ-like protein that not only couples with Gpa1 but also exhibits an ancillary role in cAMP signaling. Significantly, we discovered that Gib2 positively regulates cAMP signaling by countering the negative regulatory function of Ras1 upon Cac1 [9]. Moreover, we found that Gib2 interacts with many additional proteins such as protein kinase C (Pkc1), human intersectin homolog (Cin1), and various ribosomal subunits [9,10]. This ability of interacting with many proteins encoding important and diverse functions suggests that Gib2 could function as a critical regulator of physiology and pathogenicity in the fungus.

The human Gβ-like/RACK1 and the budding yeast *Saccharomyces cerevisiae* Asc1 proteins are important regulatory proteins for growth and differentiation that are also part of the ribosomal complex involved in ribosomal biogenesis and protein translation [11,12,13,14]. The Gib2 protein exhibits high amino acid sequence homology and certain functional similarity with Gβ-like/RACK1/Asc1 [9,10,15]. Indeed, our previous findings identified an association between Gib2 and ribosomal protein assembly [9]. To explore the global regulatory role of Gib2 in *C*. *neoformans*, we examined genome-wide targets of Gib2 by comparative proteomic analysis using two-dimensional difference gel electrophoresis (DIGE) coupled with mass spectrometry that yields novel findings.

Comparative proteomic analysis is a powerful tool that provides a qualitative and quantitative global expression profile, which is invaluable in gaining a systematic understanding of molecular processes involved in growth, development, and/or virulence [16,17]. The DIGE technique was evolved from two-dimensional electrophoresis (2-DE) that is one of the most sensitive and powerful techniques for separating hundreds of proteins [17,18]. DIGE greatly limits inter-gel variation of 2-DE and provides a wide application in proteomic studies examining changes in protein abundance, post-translational modifications, truncations and any modification that might change the size or isoelectric point of proteins [19,20]. The technique is well suited for our purposes. In the present study, we discovered that Gib2 has a global impact on protein expressions in *C*. *neoformans* as the disruption of the *GIB2* gene affected a wide array of proteins involved in various cellular processes. This includes ribosomal biogenesis, protein synthesis, stress tolerance, intracellular trafficking, amino acid and carbohydrate metabolism, and signal transduction. These findings are consistent with our proposition that Gib2 has a multifaceted regulatory role important in the growth and pathogenicity of *C*. *neoformans*.

## Materials and methods

### Fungal strains, cultures and transformation

*C*. *neoformans* var. *grubii* (serotype A) archetype H99 [21] and the derivative *gib2* mutant [9] strains were maintained on yeast peptone dextrose (YPD) agar. The *gib2* mutant strain linked to the Nourseothricin resistance marker gene (*NAT*) was described previously [9].

### Protein extraction and 2-DIGE

*C*. *neoformans* cells grown overnight in liquid YPD media at 30°C were collected, washed, and resuspended in liquid yeast nitrogen base (YNB) and grown for an additional three hours. Cells were then harvested and fragmented with glass beads (0.4–0.5 mm) using a high-speed bead-beating homogenizer (FastPrep FP120). Supernatants were recovered and crude proteins were extracted with the TCA/acetone precipitation method following the standard protocol provided by BioRad with some modifications. Briefly, cells were resuspended in 500 μl lysis buffer containing 7 M urea, 2 M thiourea, 4% (w/v) CHAPS, 65 mM DTT, and 1 mM PMSF, and fragmented for 40 x 8 sec, with 3–5 min intervals for cooling. Insoluble materials were removed by precipitation for 15 min in a microfuge. Supernatants were then mixed with 500 μl of 10% (w/v) TCA/acetone (500 μl) containing 1 mM PMSF and 0.07% (w/v) β-mercaptoethanol. Following the precipitation, the pellets were washed, resuspended in lysis buffer, and protein concentrations were estimated using the standard Bradford method [22]. For the first dimension isoelectric focusing electrophoresis, approximately 1 g of protein from each strain was labeled and co-loaded on an 18 cm, pH 3–10 nonlinear gradient IPG strip (GE Healthcare) and separated.

The second gel electrophoresis was performed using 12% SDS-PAGE. Upon completion, proteins were stained with Coomassie Brilliant Blue G-250 and images digitalized with a Typhoon image scanner. Each scan revealed one of the CyDye signals (Cy3 and Cy5). Images were analyzed with ImageQuant software (GE Healthcare Life Sciences). Protein spots were detected, matched, and normalized on the basis of the total density of gels with the parameter of percent volume, according to the software guide. For each spot, the mean relative volume (RV) was computed at every sample. The spots showing a mean RV that changed more than 1.5- fold (p < 0.05) in different stages were considered differentially expressed proteins. Protein spots of interest were picked with an Ettan Spot Picker, and identified by mass spectrometry.

### In-gel digestion, LC-MS/MS analysis, and database search

LC-MS/MS analysis, database search, and statistical analysis were provided by Appliced Biomics (Hayward, CA). Briefly, protein samples were reduced with dithiothreitol (DTT), alkylated with iodoacetamide, and digested with trypsin at 37°C overnight. 5% formic acid was added to stop the digestion and the solvent was then evaporated in a speed vacuum [9]. The dried samples were suspended in 2% acetonitrile (containing 0.1% formic acid) and subjected to LC-electrospray ionization-MS/MS analysis on a Finnigan LTQ ion trap mass spectrometer (ThermoFinnigan, San Jose, CA) as described previously [9]. Briefly, each sample was loaded into a C_18_ trap column for desalting before being eluted into a reverse-phase C_18_ analytical column for LC separation and MS detection. The acquired raw data were processed using BioWorks (version 3.3) (Thermo Electron). Following protein identification, pathway analysis and protein clustering were performed using the Database for Annotation, Visualization, and Integrated Discovery (DAVID, NIAID/NIH).

### Semi-quantitative RT-PCR

Total RNA was extracted from the wild type H99 and the *gib2* mutant strains that were subject to the same induction condition as for protein extraction, using the Trizol reagent (Invitrogen, CA). Following digestion with RNase-free DNase (RQ1, Promega), RNA was quantified using a NanoVue Plus spectrophotometer (GE Life Sciences). One μg RNA was used for reverse transcription with random hexamers (SuperScript First Strand, Invitrogen). An equal amount of cDNA (200 μg) was used in PCR with gene-specific primer pairs. Primers PW1960/PW1961, PW1962/PW1963, PW1974/PW1965, PW1966/PW1967, and PW1614/PW1615 (S1 Fig) were used to amplify partial fragments of a Hsp70-like protein (spot 12, gi5826470), flavohemoglobin (spot 36, gi37783289), a glyoxal oxidase precursor (spot 3, gi58267754), 60S ribosomal protein L9 (spot 75, gi58258095), and β-actin (*ACT*) transcripts, respectively. Images of RT-PCR stained with GelRed (Biotium) were acquired with a Bio-Rad Gel Doc XR+ System (Bio-Rad), and bands were quantified using ImageJ. The band intensity was expressed as relative absorbance units using the constitutively expressed actin gene (*ACT*) as a control for normalization of initial variations in sample concentration and for reaction efficiency. Mean and standard deviation were calculated after normalization to actin and were plotted as previously described [23].

## Results

### Identification of proteins regulated by Gib2 through a proteome approach

We previously identified Gib2 as an atypical Gβ protein that couples to Gα Gpa1 and showed that Gib2 positively regulates cAMP signaling, in conjunction with Gpa1 [9]. We also found that Gib2 interacts with approximately 50 proteins, including the protein kinase C homolog Pkc1, the endocytic adaptor protein Cin1, and several ribosomal subunits [9,10]. To further understand the global regulatory role of Gib2, we performed DIGE on protein extracts from the *gib2* mutant and the wild-type strains of *C*. *neoformans*. Since the expression of *GIB2* can be further induced upon a brief incubation in nutrient-poor YNB medium [9], we included this incubation period following overnight growth in nutrient-rich YPD in order to maximize potential differentiations between the *gib2* mutant and the wild-type strains.

**Fig 1:**
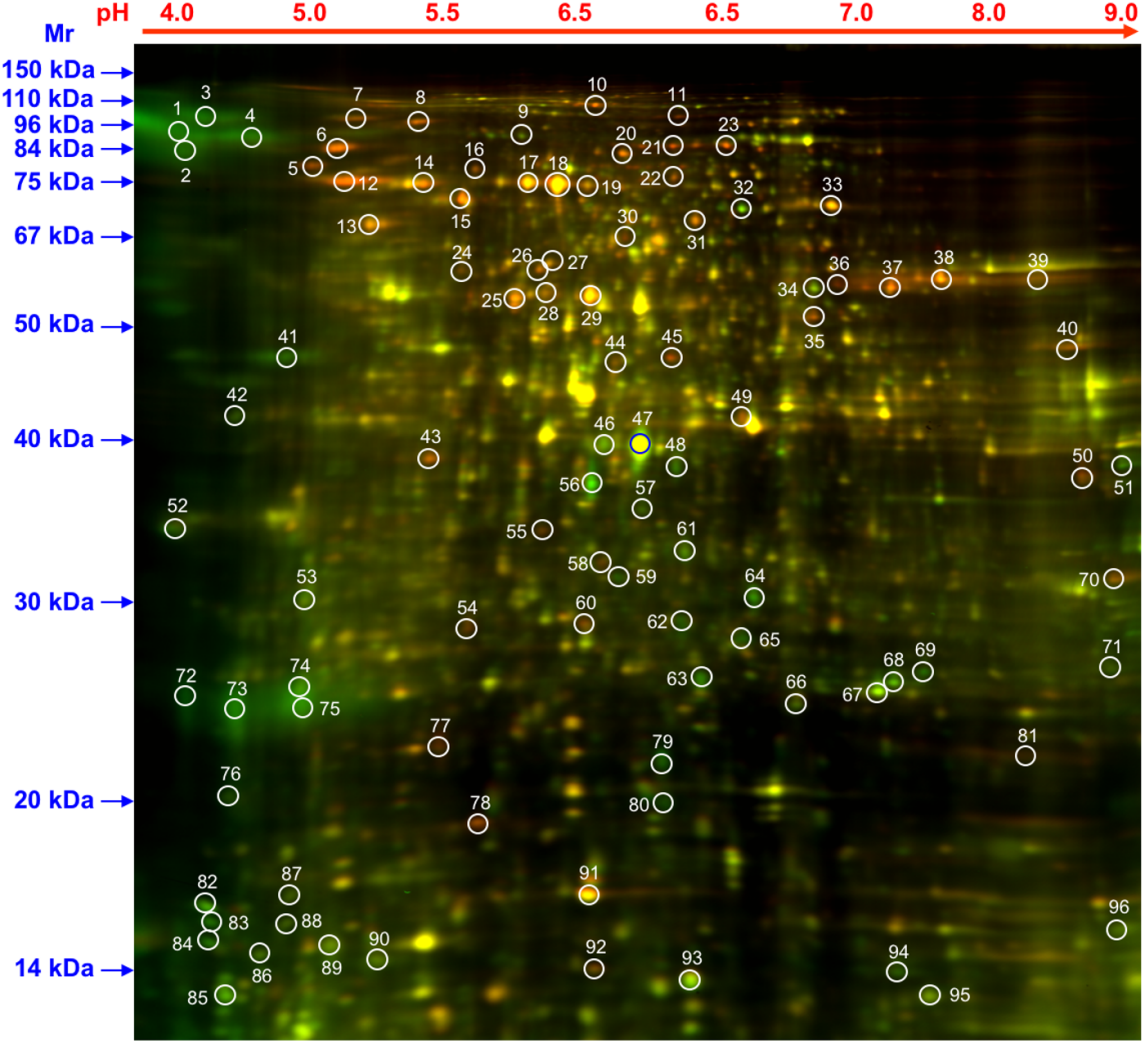
Two-dimensional gel electrophoresis (2-DIGE) of *C*. *neoformans* wild type and *gib2* mutant strains. Strains were cultured overnight in liquid YPD medium at 30°C followed by an additional three-hour incubation in YNB. Crude proteins were extracted using glass beads and a homogenizer, precipitated with TCA/acetone, normalized, labeled, and subjected to 2-DIGE. The numbered circles indicate the differentially expressed and identified protein spots.

We have obtained high quality data based on the quality of protein samples and 2-D DIGE gel runs. In total, approximately 700 protein spots could be detected reproducibly, which were distributed mostly in the pH 5–7 range and with relative molecular masses of between 14 and 110 kDa (Fig 1). Quantitative image analysis of three replicates for each sample using PDQuest 7.2 software showed a total of 96 protein spots with equal to or more than 1.5- fold difference (*P* < 0.05) in expression values in the *gib2* mutant compared to the wild-type strain H99. The 96 spots were picked and subject to mass spectrometry analysis. 75 proteins were reliably identified following searching against the *C*. *neoformans* database. For the majority of the proteins identified, experimental molecular weight (Mr) and isoelectric point (pI) match the theoretical values of the corresponding proteins. However, differences between the experimental and theoretical values of MW and pI were also noticeable (spots 3 and 4, spots 16, 17, 30 and 31). This may suggest the presence of factors such as alternate isoforms of such proteins (e.g. mRNA splice variants) or post-translational modifications.

**Fig 2:**
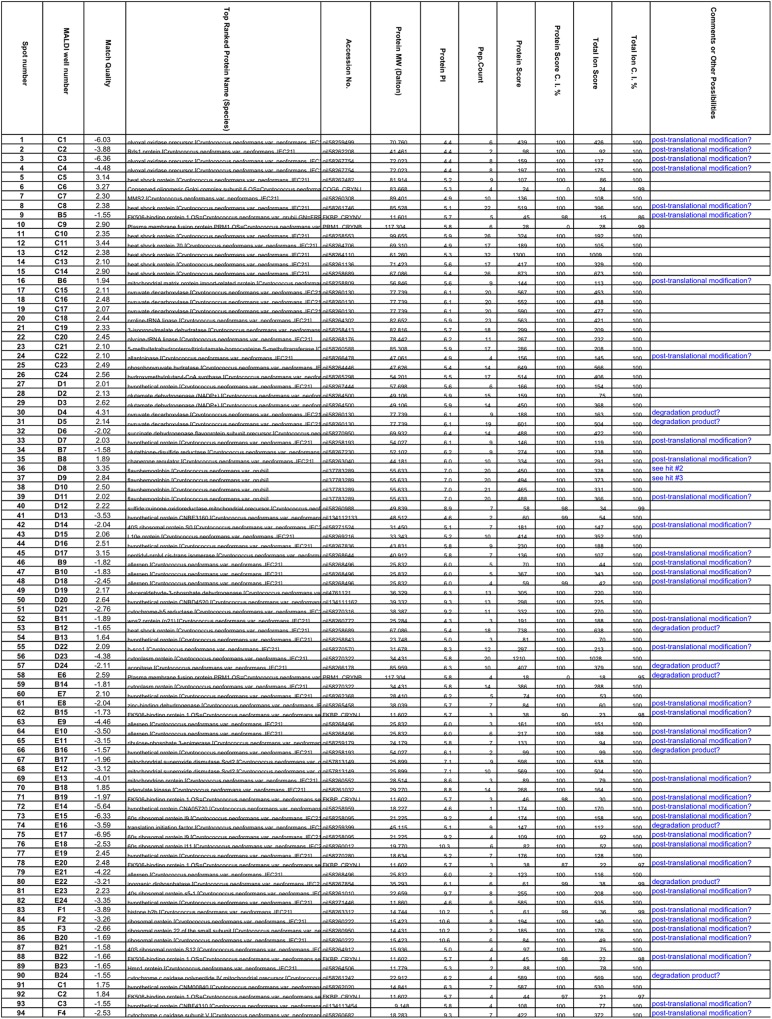
Identification of differentially expressed proteins due to *GIB2* gene disruption by MALDI-TOF-TOF MS analysis. Proteins were digested with trypsin overnight and processed protein samples were analyzed by LC-electrospray ionization-MS/MS using a Finnigan LTQ ion trap mass spectrometer [9]. The acquired raw data were processed using BioWorks (version 3.3) (Thermo Electron).

**Fig 3:**
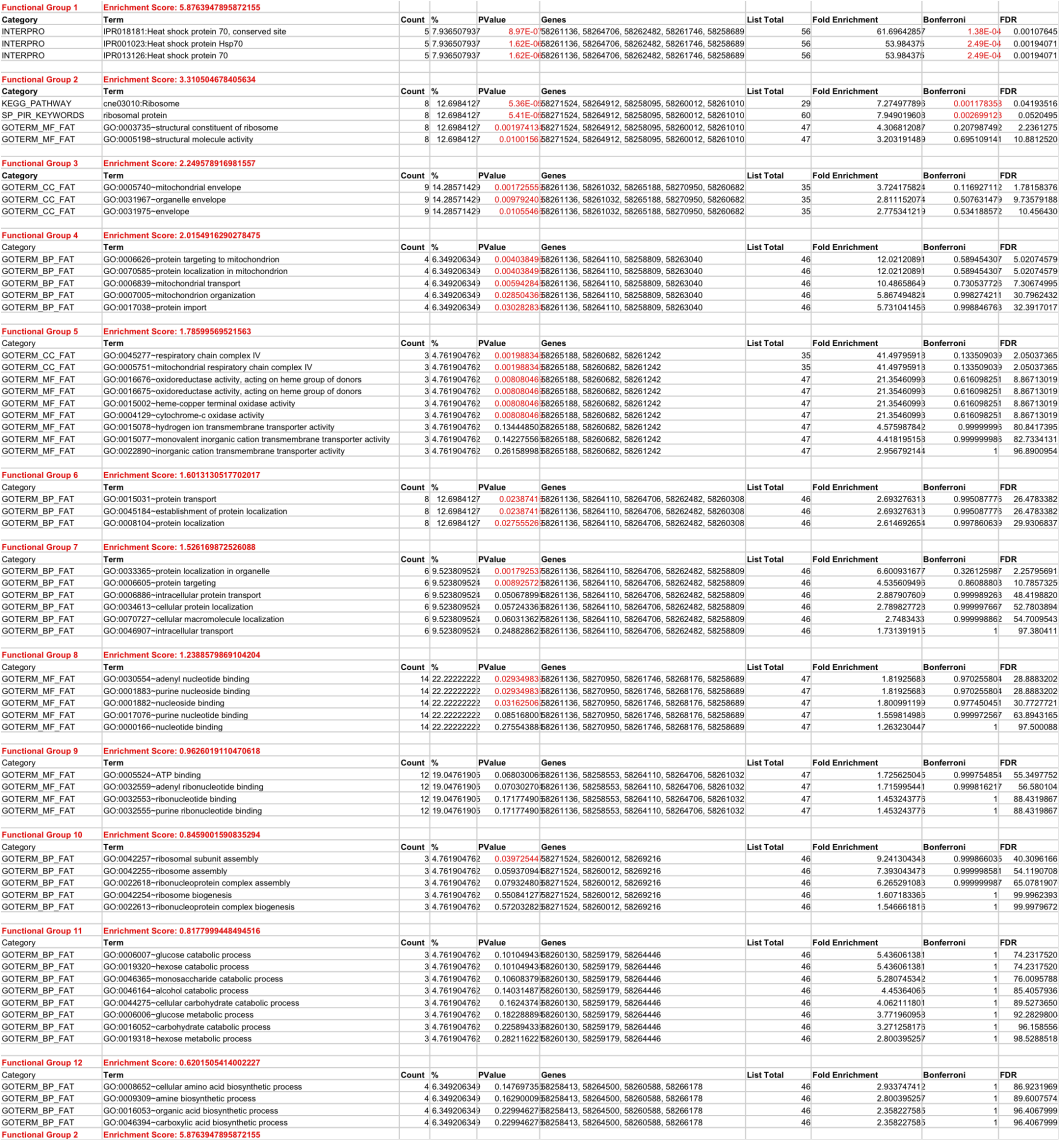
Functional groups of differentially expressed proteins due to *GIB2* disruption. The grouping was based on KEGG (http://www.kegg.jp/kegg/pathway.html) and a search of the literature (see Materials and Methods). Following protein identification, pathway analysis and protein clustering were performed using the Database for Annotation, Visualization, and Integrated Discovery (DAVID, NIAID/NIH).

Interestingly, of the 75 proteins identified, nearly the half (38) was decreased and the other half (37) increased in abundance in the *gib2* mutant (Fig 2), suggesting that the Gib2 protein has both positive and negative regulatory roles in *C*. *neoformans*. Based on their putative cellular functions, these 75 proteins were further grouped into 12 functional classes: heat shock proteins (7/75), ribosomal and ribosomal biogenesis proteins (9/75), nucleotide-binding proteins potentially implicated in signaling (5/75), energy metabolism (4/75), intracellular trafficking (11/95), carbohydrate metabolism (8/75), mitochondrial function (4/75), and amino acid metabolism (5/75). The remaining proteins were either unclassified or the function was unclear. The first eight groups have a statistical significance of p < 0.05, according to the Kyoto Encyclopedia of Genes and Genomes (KEGG) (http://www.kegg.jp/kegg/pathway.html) and search of literature (Fig 3 and S2 Fig). Overall, these findings suggest that Gib2 could modulate a variety of cellular processes and physiological characteristics, some of which are important in the growth and virulence of *C*. *neoformans*.

### Gib2 is involved in ribosomal biogenesis and function

In previous studies, we identified several proteins involved in ribosomal biogenesis and protein translation through GST-Gib2 affinity purification coupled with mass spectrometry [9]. One of these proteins was the eukaryotic initiation factor-4A (eIF4A) homolog that is involved in binding of mRNA to 40S ribosomal subunits (translation initiation) and unwinding of double-stranded RNA (splicing). In that study, we found that the expression of eIF4A was reduced by 3.6- fold in the *gib2* mutant [9]. We also provided structural based evidence pinpointing the interaction between Gib2 and eIF4A [15]. Consistent with these early observations, we found that a large number of differentially expressed proteins were either ribosomal components or proteins associated with ribosomal function (Fig 2). The abundance of ribosomal and ribosomal biogenesis associated proteins (11) was remarkably changed in the *gib2* mutant, in comparison to that in the wild type H99 strain. Among these proteins, nine were decreased in expression, and only two (gi 58261010 and gi 58269216) were increased (Fig 2). Among those decreased, the putative 60S ribosomal protein L9 (gi 58258095) appears to be the most affected with reductions of 6.3- to 7- fold.

**Fig 4:**
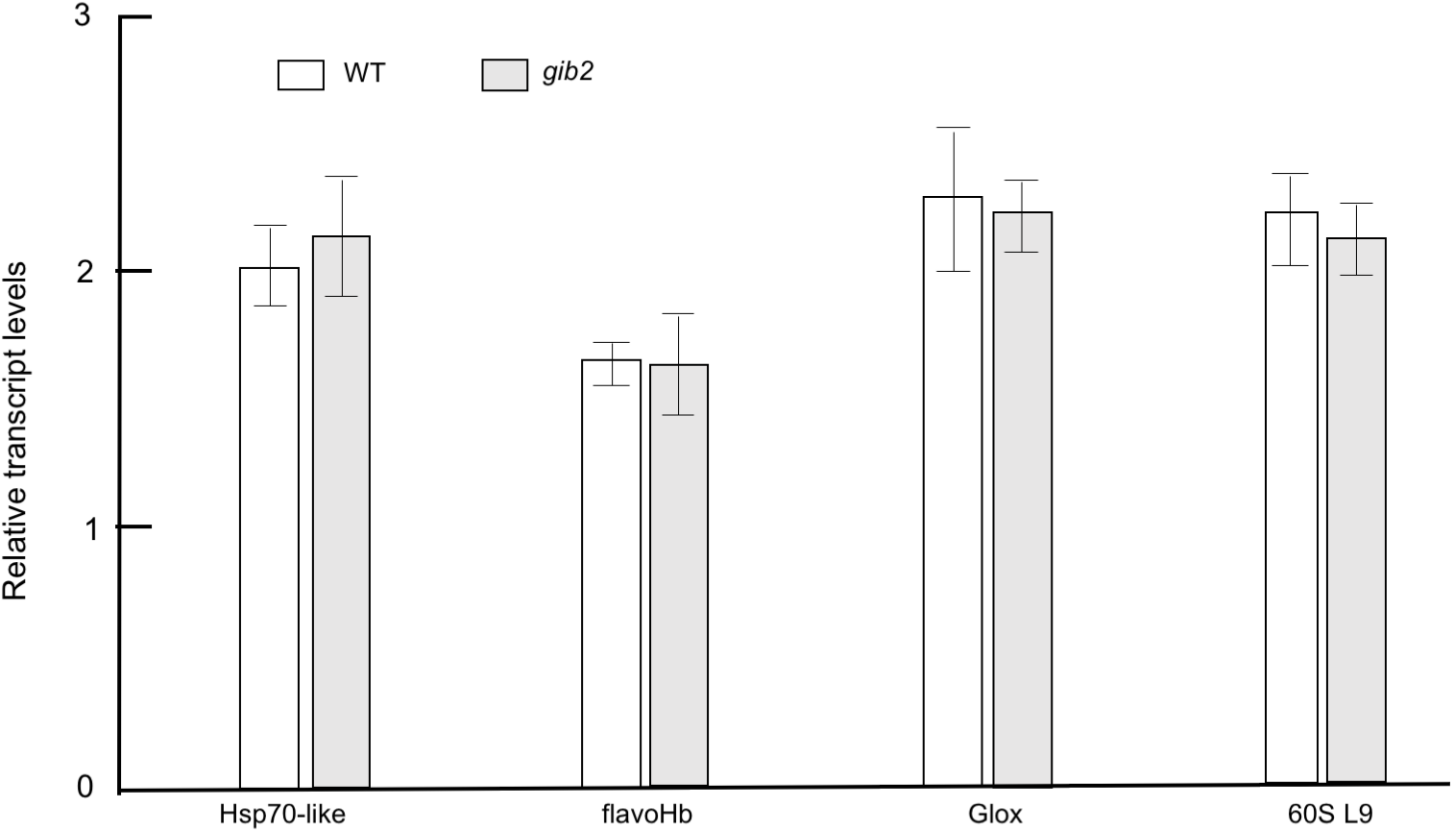
Differences in expressions of four representative genes are largely similar between the wild type and the *gib2* mutant strains. The induction time for cells in YNB was 3 hours. RT-PCR was repeated twice and mean values were used to calculate the expression rate relative to that of the *ACT* gene encoding actin with error bars (average + SD) shown. PCR cycles were limited to 25. Hsp70-like, flavoHb, Glox, and 60S L9 denote, respectively, a Hsp70 protein homolog (spot 12, gi 5826470), flavohemoglobin (spot 36, gi 37783289), a glyoxal oxidase precursor (spot 3, gi 58267754), and 60S ribosomal protein L9 (spot 75, gi 58258095).

To investigate if the changes in protein expression were rather due to mRNA abundance changes than modulated protein translation, we opted to use the semi-quantitative RT-PCR approach to examine the expression of four selected genes that correspond to proteins with the most changes. The Hsp70-like protein (spot 12, gi 5826470) and flavohemoglobin (spot 36, gi 37783289) were increased by at least 3 folds in the *gib2* mutant, whereas the glyoxal oxidase precursor (spot 3, gi 58267754) and 60S ribosomal protein L9 (spot 75, gi 58258095) were decreased in expression by at least 6- folds. However, as revealed by semi-quantitative RT-PCR analysis, the expression of these genes was largely similar between both strains. Relative abundance of Hsp70, flavohemoglobin, glyoxal precursor, and 60S ribosome L9 are 2.0± 0.2, 1.7±0.1, 2.2±0.4, and 2.3±0.2 in H99 versus 2.3±0.4, 1.6±0.3, 2.1±0.2, and 2±0.2 in the *Gib2* mutant (Fig 4). Collectively, these findings suggest that Gib2 participates in ribosomal biogenesis and protein translation.

### Gib2 has roles in stress responses

The 70-kilo Dalton heat shock proteins (Hsp70 or DnaK) are a family of highly conserved, ubiquitously expressed proteins that chaperone the folding of a large variety of proteins. Hsp70 proteins also help to protect cells from the stress caused by hyperthermia, oxidants, or changes in pH (reviewed in [24,25]). Our DIGE data indicated that *C*. *neoformans* could encode at least six Hsp70 and one Hsp71 protein homologs, whose translation are all subject to regulation by Gib2. Indeed, all these proteins were increased in abundance by 2.1- to 3.4- fold. The expression of Ssa1, one of the Hsp70 proteins containing a DEAD domain and is involved in the stress response [26,27] was mostly increased by 3.4- fold. Given the established roles of Hsp70 proteins, the increased expression of Hsp70/71 proteins may functionally compensate for a lack of Gib2 adaptor/ chaperon function needed for nascent protein synthesis, folding, and maturation.

### Gib2 has roles in intracellular trafficking

In a previous study, we found that Gib2 interacts with Cin1, a homolog of human intersectin ITSN1 protein, in *C*. *neoformans* [10]. The Cin1 protein shares a high amino acid sequence homology with the mammalian intersectin ITSN1 protein [28]. We subsequently demonstrated Cin1 as an endocytic adaptor protein that has a pleiotropic function important for endocytosis, actin cytoskeleton dynamics, and virulence [29]. The current 2-DIGE study results support the involvement of Gib2 in Cin1-regulated intracellular trafficking. Indeed, as indicated by our current findings, the expression of eight putative intracellular trafficking proteins was differentially affected due to the *GIB2* gene disruption. As shown in Fig 2, the expression of plasma fusion protein Prm1 OS (Prm1_Crynb) showed a 2.6- fold of increase in the *gib2* mutant strain. Similarly, the expression of a conserved oligomeric Golgi complex subunit 6 (Cog6_Crynj) putatively involved in ER to Golgi vesicle transport was increased by 3.3- fold because of *GIB2* gene disruption (Fig 2).

## Discussion

The human fungal pathogen *Cryptococcus neoformans* remains a significant cause of morbidity and mortality in individuals with compromised immune status, especially in regions of high HIV prevalence and limited healthcare resources [30]. Upon inhalation, the fungus exhibits a predilection for the human central nervous system where it can cause potentially fatal meningoencephalitis if untreated. Virulence of the fungus is a multifaceted and attributable to multiple and diverse traits. Among those of pathogenic-intrinsic origin, signal transduction pathways are means by which the fungus senses and responds to environmental stimuli and produces virulence factors. In *C*. *neoformans*, Gpa1- and Gpb1*-*mediated G-protein signal transduction pathways, as well as additional signaling molecules or pathways, are important in the modulation of growth and pathogenesis (reviewed in [2,31]).

Our previous studies established that Gib2 participates in Gpa1 signaling as an atypical Gβ-like protein through coupling with Gpa1 and Gpg1/Gpg2 as a heterotrimeric complex, and Gib2 also positively promotes cAMP signaling independent of Gpa1 [10]. These studies demonstrated that Gib2 is a critical component of overall signal transduction pathways required for normal growth and virulence of *C*. *neoformans*. Importantly, Gib2 interacts with at least 47 additional proteins with diverse functions, indicating its capacity as a regulatory scaffold protein to modulate multiple protein-protein interactions, similar to Gβ-like/RACK1/Asc1 family proteins of other organisms. To examine this multifaceted role at the genome-wide level, we performed a protein expression profiling study by 2D-DIGE coupled with mass spectrometry analysis. Using pathway analysis of predicted proteins identified in the Protein ID report, we clustered 76 proteins into 12 functional protein groups. Among the groups, ribosomal subunits, ribosomal biogenesis components, intracellular trafficking, and signaling components are included in the top of the eight groups. Together with our previous findings, this new round of findings further established crucial function of Gib2 in growth and pathogenicity [9]. Significantly, these findings also suggest that Gib2 represents an important therapeutic target.

Our finding is highly accordant with those of human and *S*. *cerevisiae* studies indicating Gβ-like/RACK1 and Asc1 proteins are ribosomal core proteins [10,11,12,13,14,15]. In these studies, Gβ-like/RACK1 and Asc1 were found to interact with the 40S ribosome subunit largely via the RDK (Arg36-Asp37-Lys38) amino acid residues in the first WD40 domain. Moreover, Gβ-like/RACK and Asc1 associate with the head region of the 40S ribosomal subunit in the vicinity of the mRNA exit channel [32,33]. Gib2 also contains the exact RDK residues in its sequence [9]. LACK1, a RACK/Asc1/Gib2 homolog in the parasite *Leishmania major* contains the conserved RDK/G residues and was located in the polysome (polyribosome) complex [34]. In addition, the eukaryotic initiation factor 4A (eIF4A) is required for the binding of mRNA to 40S ribosomal subunits (translation initiation) and unwinding double-stranded RNA (splicing). In *Leishmania*, the parasite cells deficient in LACK were more susceptible to hippuristanol, an eIF4A-specific inhibitor, than WT control strains [34]. Using GST-tagged protein purification and co-immunoprecipitation approaches, we earlier demonstrated that Gib2 interact with eIF4A [9]. We further mapped out the Gib2-eIF4A interaction based on the analysis of the Gib2 crystal structure [15]. Although not demonstrated in this study, the expression of eIF4A was reduced by 3.6- fold in the *gib2* mutant, supporting that Gib2 has a significant role in the initiation of protein translation.

Eukaryotic Hsp70/71 proteins are involved in the modulation of nascent protein folding and processing, thereby protecting cells from the stress. The findings that Gib2 exerts a strong influence over the expression of all Hsp70/71 homologs may suggest their elevated expression is a compensatory effect made by in the *gib2* mutant cells (or that loss of Gib2 induces cellular stress). Overexpression of Hsp70/71 proteins may compensate for the lack of Gib2 in protein translation. One of the resulting functions of increased Hsp70 proteins is that they provide a means of protection against stresses, whether it is hyperthermia, the oxidative stress, or host-mediated stress conditions.

Finally, given the importance of Gib2 in modulating signaling pathways, we failed to identify relevant proteins in DIGE coupled with mass spectrometry. We hypothesized that this may be due to the nature of regulatory proteins whose expressions may be highly regulated either spatially or temporally and that our sampling method failed to meet these specific conditions. Nevertheless, by employing the genome-wide global approach, we gained further insights into the complex function and pathway of Gib2 biology that is important for morphogenesis, development, and pathogenicity. Significantly, this approach has allowed for the simultaneous examination of multiple proteins subjected to translational regulation by Gib2. Further examination of these proteins and the pathways they represent are likely to expose more potential targets for therapeutic intervention of infection caused by *C*. *neoformans*.

## Supporting information

S1 Fig Primers used in this study. (TIFF)

S2 Fig MALDI-TOF-TOF MS analysis of differentially expressed proteins in *C*. *neoformans*. (XLS)
