# Supplementary figures and images for: Comparative proteomic analysis of Gib2 validating its adaptor function in Cryptococcus neoformans
Source: PLoS One. 2017 Jul 7;12(7):e0180243. doi: 10.1371/journal.pone.0180243 (PMC5501510; doi:10.1371/journal.pone.0180243)

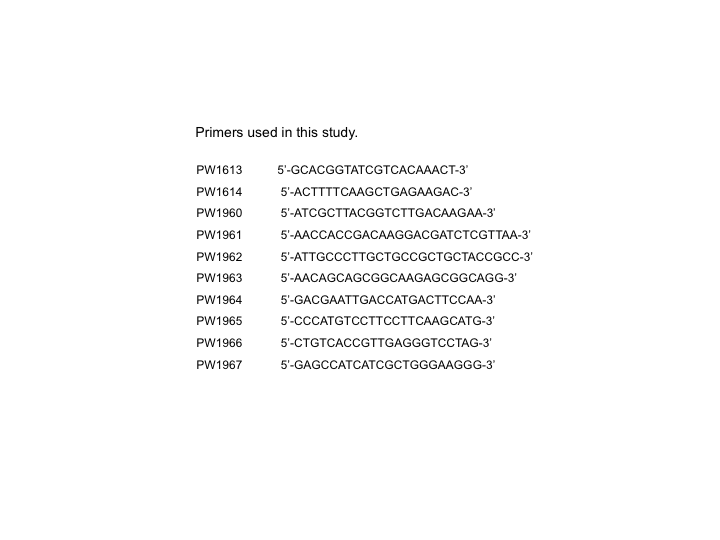

Supplement: S1 Fig — (TIFF) [file pone.0180243.s001.tiff]
